# Akt Phosphorylation of Hepatitis C Virus NS5B Regulates Polymerase Activity and Hepatitis C Virus Infection

**DOI:** 10.3389/fmicb.2021.754664

**Published:** 2021-10-22

**Authors:** Rosario Sabariegos, Laura Albentosa-González, Blanca Palmero, Pilar Clemente-Casares, Eugenio Ramírez, Carlos García-Crespo, Isabel Gallego, Ana Isabel de Ávila, Celia Perales, Esteban Domingo, Antonio Mas

**Affiliations:** ^1^Centro Regional de Investigaciones Biomédicas, Universidad de Castilla-La Mancha, Albacete, Spain; ^2^Facultad de Medicina, Universidad de Castilla-La Mancha, Albacete, Spain; ^3^Unidad de Biomedicina UCLM-CSIC, Madrid, Spain; ^4^Facultad de Farmacia, Universidad de Castilla-La Mancha, Albacete, Spain; ^5^Centro de Biología Molecular “Severo Ochoa” (CBMSO) (CSIC-UAM), Consejo Superior de Investigaciones Científicas (CSIC), Madrid, Spain; ^6^Centro de Investigación Biomédica en Red de Enfermedades Hepáticas y Digestivas (CIBERehd) del Instituto de Salud Carlos III, Madrid, Spain; ^7^Department of Clinical Microbiology, Instituto de Investigación Sanitaria-Fundación Jiménez Díaz University Hospital, Universidad Autónoma de Madrid (IIS-FJD, UAM), Madrid, Spain

**Keywords:** HCV (hepatitis C), NS5B (non-structural protein) polymerase, Akt, virus replication, phosphorylation

## Abstract

Hepatitis C virus (HCV) is a single-stranded RNA virus of positive polarity [ssRNA(+)] that replicates its genome through the activity of one of its proteins, called NS5B. This viral protein is responsible for copying the positive-polarity RNA genome into a negative-polarity RNA strand, which will be the template for new positive-polarity RNA genomes. The NS5B protein is phosphorylated by cellular kinases, including Akt. In this work, we have identified several amino acids of NS5B that are phosphorylated by Akt, with positions S27, T53, T267, and S282 giving the most robust results. Site-directed mutagenesis of these residues to mimic (Glu mutants) or prevent (Ala mutants) their phosphorylation resulted in a reduced NS5B *in vitro* RNA polymerase activity, except for the T267E mutant, the only non-conserved position of all those that are phosphorylated. In addition, *in vitro* transcribed RNAs derived from HCV complete infectious clones carrying mutations T53E/A and S282E/A were transfected in Huh-7.5 permissive cells, and supernatant viral titers were measured at 6 and 15 days post-transfection. No virus was rescued from the mutants except for T53A at 15 days post-transfection whose viral titer was statistically lower as compared to the wild type. Therefore, phosphorylation of NS5B by cellular kinases is a mechanism of viral polymerase inactivation. Whether this inactivation is a consequence of interaction with cellular kinases or a way to generate inactive NS5B that may have other functions are questions that need further experimental work.

## Introduction

Hepatitis C virus (HCV) is a positive single-stranded RNA virus [ssRNA(+)] belonging to Flaviviridae family. HCV genome replication takes place in replication complexes where the non-structural NS5B protein produces positive single-stranded genome copies [RNA(+)] through an intermediary of negative polarity [RNA(−)] (Neufeldt et al., 2018; Tabata et al., 2020). The HCV protein NS5B is an RNA-dependent RNA polymerase (RDRP) which, like other polymerases in its class, shows a structure that has been compared to the shape of a right hand with three subdomains called fingers, palm, and thumb (Sesmero and Thorpe, 2015). The subdomain of the palm comprises three well-preserved motifs A (D^220^-X(4)-D^225^), B (S^282^-X(8)-N^291^), and C (G^317^D^318^D^319^), which define the catalytic center. The aspartic acid residues D^220^ in motif A and D^318^ and D^319^ in motif C are involved in the coordination of the divalent cations (Mg^2+^ and/or Mn^2+^) essential for the formation of the phosphodiester bond. Residues D^225^ of motif A, and S^282^ and N^291^ residues of motif B are involved in selection of ribonucleoside triphosphates over dNTPs and, thus, determine whether RNA rather than DNA is synthesized (Sesmero and Thorpe, 2015).

Viruses are obligate intracellular parasites that usurp the cellular machinery to complete their replicative cycle. This means that viral components need to interact with cellular components to direct cellular activity according to viral interests. HCV is no exception, and an enormous amount of interactions between viral components and cellular proteins have already been described (de Chassey et al., 2008; Dolan et al., 2013; Hagen et al., 2014). HCV proteins NS3, NS5A, and core have shown the most extensive network of interactions with host factors (de Chassey et al., 2008). NS5B also interacts with cellular proteins (Hamamoto et al., 2005; Munakata et al., 2005; Watashi et al., 2005; Kusakawa et al., 2007; Inoue et al., 2011; Hillung et al., 2012), and some of its interactions require partial denaturation of NS5B and subsequent loss of RDRP activity. For example, the retinoblastoma tumor suppressor is down regulated by interacting with NS5B via amino acids located in the catalytic center (C motif) (Munakata et al., 2005). For this interaction to occur, the NS5B protein must expose amino acids located in the active center. These data indicate that an inactive NS5B may be modulating an important surveillance pathway.

We have previously described the interaction of NS5B with the cellular kinase Akt and the changes in subcellular localization related with this protein:protein interaction (Valero et al., 2016). Furthermore, we documented that Akt phosphorylates NS5B and that inhibitors of Akt affect HCV replication in cell culture (Valero et al., 2016). Viral RNA polymerase phosphorylation has also been described in other viral systems previously (Barik et al., 1995; Jakubiec et al., 2006; Schmid et al., 2007), and norovirus RNA polymerase is phosphorylated by Akt as well (Eden et al., 2011). The PI3K-Akt pathway, transiently activated during HCV entry (Liu et al., 2012; Qian et al., 2020), has been linked to HCV infection and related metabolic disorders (Qadri et al., 2012; Zhang et al., 2018). Therefore, modulation of Akt and Akt-related proteins could be of great importance for HCV replication.

In this study, we describe the positions of the HCV NS5B protein that are phosphorylated by the cellular kinase Akt. Furthermore, we show that mutants mimicking phosphorylation at these positions lead to proteins with very low RDRP activities. In addition, viruses carrying mutations mimicking Akt phosphorylation of T53 or S282 residues were unable to replicate in cultured cells. Akt:NS5B interaction produces an inactive viral polymerase whose role needs to be elucidated.

## Materials and Methods

### Reagents, Expression Plasmids, and Inhibitors

Plasmid pET_NS5BΔ21 encoding HCV NS5B from strain HC-J4 with a 21 amino acid deletion at the C-terminal end has been described previously (Lopez-Jimenez et al., 2014). Recombinant Akt/PKB was purchased from Biaffin (PK-PKBA-020, Biaffin GmbH&Co), and lipofectamine 2000 from Invitrogen. Huh7.5 cells were kindly provided by Dr. R. Bartenschlager (University of Heidelberg, Germany).

### Hepatitis C Virus NS5B WT and Mutants Purification

Point mutants in NS5B were generated by site-directed mutagenesis following the manufacturer’s instructions (QuikChange Site-Directed Mutagenesis, Agilent Technologies). Synthetic oligonucleotides used for point mutant generation are described in Table 1. NS5B wild type and mutants were over-expressed and purified as described previously (Lopez-Jimenez et al., 2014; Valero et al., 2016).

**TABLE 1:** Oligonucleotides used in this study.

Name	Sequence (5′–>3′)
pJ4 S27A s	gcccatcaacccgttggccaactctttgctgcgt
pJ4 S27A as	acgcagcaaagagttggccaacgggttgatgggc
pJ4 S29A s	aacccgttgagcaacgctttgctgcgtcacc
pJ4 S29A as	ggtgacgcagcaaagcgttgctcaacgggtt
pJ4 S27A/S29A s	cccatcaacccgttggccaacgctttgctgcgtcacc
pJ4 S27A/S29A as	ggtgacgcagcaaagcgttggccaacgggttgatggg
pJ4 T53A s	ccggcagaagaaggtcgcctttgacagattgca
pJ4 T53A as	tgcaatctgtcaaaggcgaccttcttctgccgg
pJ4 T267A s	cgggggtcccctggctaactcaaaaggg
pJ4 T267A as	cccttttgagttagccaggggacccccg
pJ4 T269A s	gggtcccctgactaacgcaaaagggcagaactg
pJ4 T269A as	cagttctgcccttttgcgttagtcaggggaccc
pJ4 T267A/T269A s	cgggggtcccctggctaacgcaaaagggcagaa
pJ4 T267A/T269A as	ttctgcccttttgcgttagccaggggacccccg
pJ4 T282A s	cgccggtgccgcgcagctggcgtgc
pJ4 T282A as	gcacgccagctgcgcggcaccggcg
pJ4 S27E s	ctgcccatcaacccgttggagaactctttgctgcgtcac
pJ4 S27E as	gtgacgcagcaaagagttctccaacgggttgatgggcag
pJ4 S29E s	ccatcaacccgttgagcaacgagttgctgcgtcaccacaacat
pJ4 S29E as	atgttgtggtgacgcagcaactcgttgctcaacgggttgatgg
pJ4 S27E/S29E s	agtaagctgcccatcaacccgttggagaa cgagttgctgcgtcaccacaacatggtc
pJ4 S27E/S29E as	gaccatgttgtggtgacgcagcaa ctcgttctccaacgggttgatgggcagcttact
pJ4 T282E s	tatcgccggtgccgcgcagagggcgtgctgacg
pJ4 T282E as	cgtcagcacgccctctgcgcggcaccggcgata
pJ4 T53E s	ggacttgcaatctgtcaaactcgaccttcttctgccggagg
pJ4 T53E as	cctccggcagaagaaggtcgagtttgacagattgcaagtcc
pJ4 T267E/T269E s	ggctttacatcgggggtcccctggaga acgagaaagggcagaactgcggttatcg
pJ4 T267E/T269E as	cgataaccgcagttctgccctttctcgttctccaggggacccccgatgtaaagcc
pJ4 T267E s	cagttctgcccttttgagttctccaggggacccccgatgtaaa
pJ4 T267E as	tttacatcgggggtcccctggagaactcaaaagggcagaactg
pJ4 T269E s	gcagttctgccctttctcgttagtcaggggacccccga
pJ4 T269E as	tcgggggtcccctgactaacgagaaagggcagaactgc
NS5B T53E s	tcacagagggctaaaaaggtagagtttgacaggacgcaagtgctc
NS5B T53E as	gagcacttgcgtcctgtcaaactctacctttttagccctctgtga
NS5B T282E s	agacgttgccgcgccgagggggtgctaaccact
NS5B T282E as	agtggttagcaccccctcggcgcggcaacgtct
NS5B T53A s	cagagggctaaaaaggtagcttttgacaggacgcaag
NS5B T53A as	cttgcgtcctgtcaaaagctacctttttagccctctg
NS5B T282A s	acgttgccgcgccgccggggtgctaacc
NS5B T282A as	ggttagcaccccggcggcgcggcaacgt

### *In vitro* Kinase Assay

Kinase assays were performed as previously described (Albentosa-Gonzalez et al., 2021). Briefly, HCV NS5B (3 μg) was incubated in 20 mM Hepes pH 7.4, 10 mM MgCl_2_, 10 mM MnCl_2_, 1 μCi of γ[^32^P]-ATP, 1 mM DTT, in the presence of 0.5 μg of recombinant Akt/PKB (PK-PKBA-A020, Biaffin GmbH&Co). Following SDS-PAGE electrophoresis, the gel was dried and exposed to phosphorimager screens, and scanned with Typhoon 9600 (Molecular Dynamics) to detect radiolabeled products.

### In-Gel Digestion and Reverse Phase-Liquid Chromatography RP-LC-MS/MS Analysis

The identification of phosphorylated NS5B residues was performed as previously described (Albentosa-Gonzalez et al., 2021). Briefly, phosphorylated protein was digested *in situ* with sequencing grade trypsin (Promega, Madison, WI, United States) and analyzed by RP-LC-MS/MS in an Easy-nLC II system coupled to an ion trap LTQ-Orbitrap-Velos-Pro mass spectrometer (Thermo Scientific). The MS/MS spectra from the peptides were analyzed by assigning the fragment ions to the candidate sequence after calculating the series of theoretical fragmentations.

### *In vitro* RNA-Dependent RNA Polymerase Replication Assays

RNA polymerase assays were performed using the symmetric substrate LE-19, which is capable of *de novo* initiation (DN) and primer-extension (PE), as previously described (Clemente-Casares et al., 2011; Lopez-Jimenez et al., 2014). 200 nM NS5B was pre-incubated for 30 min in a reaction mixture containing 20 mM MOPS, pH 7.3, and 5 mM MnCl_2_. Reactions were started by adding 500 μM GTP, 100 μM ATP, and UTP, and 1 μCi α[^32^P]CTP (3000 Ci mmol, PerkinElmer Life Sciences). Reactions were stopped by adding EDTA/formamide loading buffer at different time points as indicated. Products were separated using denaturing polyacrylamide (23% PAA, 7 M urea) gel electrophoresis. Gels were exposed to phosphorimager screens and scanned with Typhoon (Molecular Dynamics). Quantification was achieved by running samples on parallel gels and determining band volumes using ImageQuant software (GE Healthcare).

### Cell Culture Virus Infection

The origin of the Huh-7.5 cell line, procedures for cell growth in Dulbecco’s modified Eagle’s medium (DMEM), the virus used in the experiments rescued from plasmid Jc1FLAG2(p7-nsGluc2A) (a chimera of J6 and JFH-1 from genotype 2a), and the procedures used to prepare the initial virus stock HCVp0, to titrate viral infectious particles, and to quantify viral RNA have been described previously (Perales et al., 2013; Sheldon et al., 2014). To perform infections for immunofluorescence and RNA quantification assays, 1 × 10^5^ Huh-7.5 cells were infected with HCVp0 at a multiplicity of infection (MOI) of 0.5 Tissue Culture Infectious Dose (50%) TCID50/cell. The infected cells were further incubated at 37°C for 6 and 15 days. Absence of contamination was checked by maintaining and titrating mock-infected cells and their supernatants in parallel with the infected cultures. No infectivity in the mock-infected cultures was detected in any of the experiments.

Plasmid Jc1FLAG2(p7-nsGluc2A) was used as a template for constructing T53A, T53E, S282A, and S282E mutants by site-directed mutagenesis using the oligonucleotides described in Table 1. Plasmids carrying the selected mutations were transcribed *in vitro* and the genomic RNA from WT or mutant virus was used to transfect cells as described above to produce HCVp0 virus stock. The supernatant from these transfections was used to titer the virus obtained. Supernatant from infections with mutant virus in which viral titer was obtained was used to purify viral RNA that was retrotranscribed, PCR amplified, and sequenced to test for mutation reversal.

## Results

### Phosphoproteomic Studies

**FIGURE 1:**
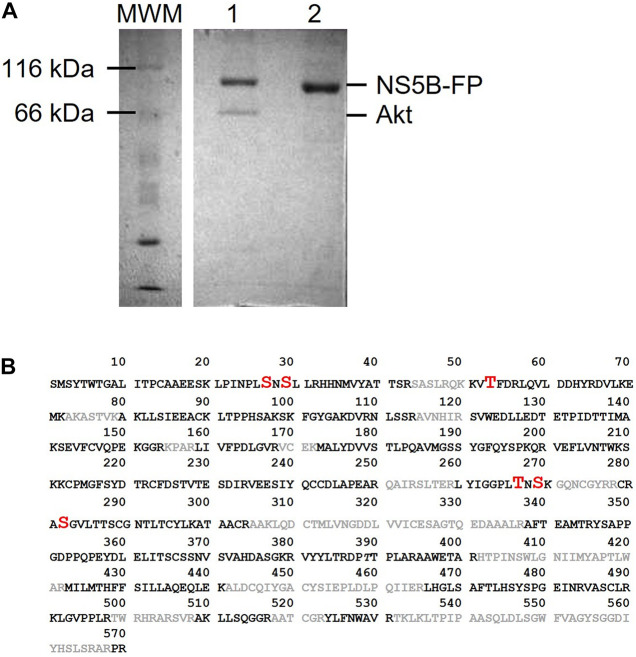
**(A)** SDS-PAGE gel showing products obtained after Akt phosphorylation of NS5B-FP (lane 1). Unphosphorylated NS5B-FP is shown in lane 2. Molecular weight marker (MWM) is on the left of the gel and sizes for NS5B-FP and Akt are indicated on the right. **(B)** Amino acid sequence of HCV NS5B from strain HC-J4. Phosphorylated sites identified in phosphoproteomics are in red (with increased font size). Sequence uncovered by the phosphoproteomic analysis is in gray color.

Previous results from our laboratory have shown that the Akt protein phosphorylates the NS5B polymerase of HCV (Valero et al., 2016). To analyze the effect that this phosphorylation causes in the biology of the virus, we first wanted to identify the residues phosphorylated by this Ser/Thr kinase. To this end, we performed an *in vitro* Akt phosphorylation assay using recombinant protein NS5B (66 kDa) fused to the fluorescent protein EGFP (NS5B-FP) and γ[^32^P]-ATP as substrates. The fused protein shows a molecular mass of approximately 95 kDa that can easily be distinguished from Akt (68 kDa). The products of the phosphorylation reaction were resolved by SDS-PAGE and two bands were detected, one at about 100 kDa corresponding to NS5B-FP, and the other at about 65 kDa corresponding to the autophosphorylated Akt (Figure 1A). The phosphorylation experiment was repeated with cold ATP for proteomic purposes. The NS5B-FP band was extracted and digested with trypsin, yielding a sequence coverage of 70% in the proteomic analysis (Figure 1B).

**FIGURE 2:**
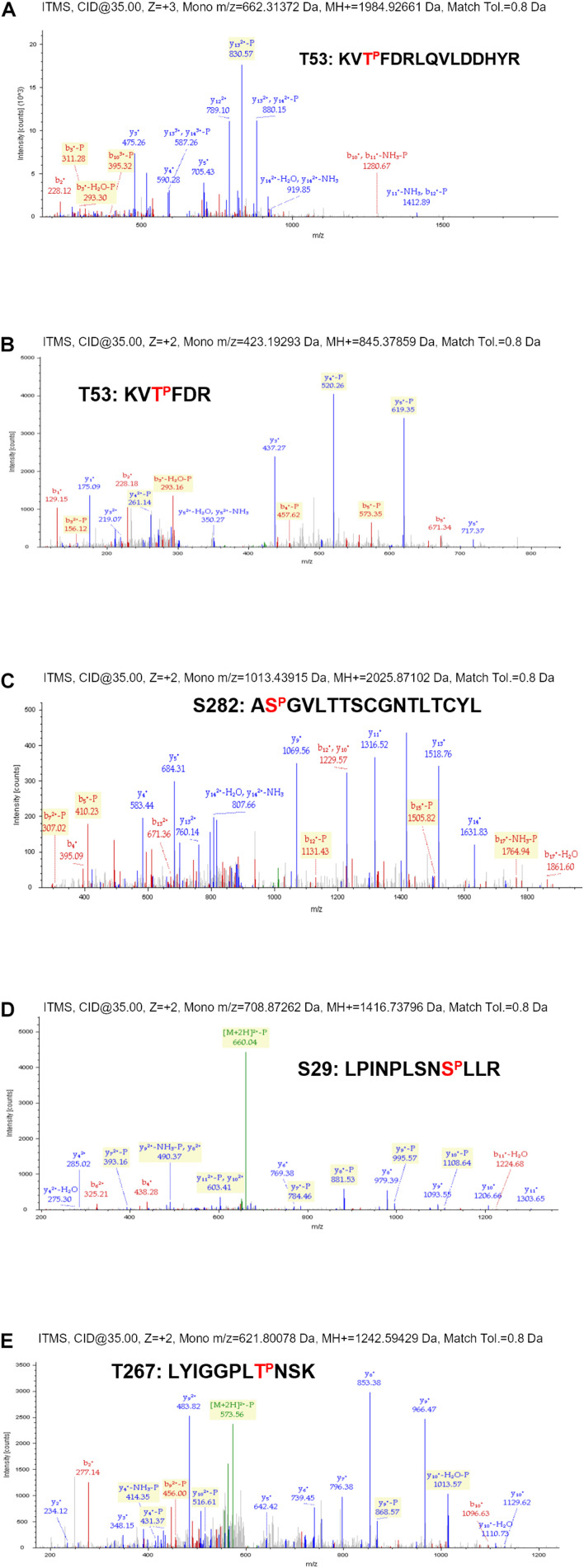
Phosphoproteomic details from the phosphorylation experiment described in Figure 1. MS/MS profiles for NS5B-FP tryptic peptides encompassing residues T53 **(A,B)**, S282 **(C)**, S29 **(D)**, and T267 **(E)**. Peptide sequences corresponding to each spectrum are indicated with the identified phosphorylated residue marked in red.

**FIGURE 3:**
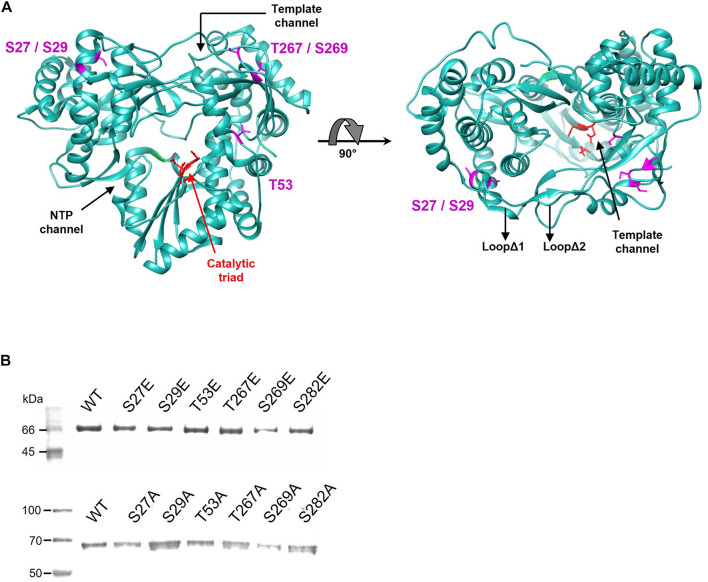
**(A)** Localization of the residues phosphorylated by Akt. The positions of the amino acids identified by proteomics are shown in magenta on the structure of the NS5B HC-J4 (PDB: 1NB4) protein. The amino acids of the catalytic triad are shown in red. The most important structural elements (template channel, nucleotide entry channel and loops Δ1 and Δ2) are also indicated. **(B)** SDS-PAGE gel showing purified WT and Glu (upper panel) or Ala (lower panel) mutant proteins for each position. The molecular weight marker is shown on the left. The identity of the protein in each lane is indicated above the protein bands.

Proteomic analysis of the trypsin digestion products allowed the identification of the phosphorylated residues S29, T53, T267, and S282 (Figure 2). Ser29 is located in the subdomain called fingertips, and is involved in the interaction with the thumb (Figure 3A). Residues T53 and T267 are located in the fingers subdomain, and are involved in the helix-helix interactions that stabilize this subdomain (Figure 3A). Finally, the S282 residue is part of the motif B site and interacts with the D225 residue of motif A for the correct positioning of the NTP during the formation of the phosphodiester bond. Two other residues (S27 and S269) could be phosphorylated and were not well identified because of their proximity to the most likely ones (S29 and T267). In any case, it is much less likely that they are phosphorylated and this was the reason they were not included in some experiments.

### NS5B Activity Studies

Next, we sought to determine the effect of the phosphorylation of the residues described above on the RDRP activity of the NS5B protein. To this end, mutations that mimic phosphorylated Ser or Thr residues at these positions were introduced by site-directed mutagenesis, yielding proteins carrying the mutations S27E, S29E, T53E, T267E, S269E, and S282E. We also generated mutants carrying Ala in these positions. All these proteins were overexpressed in *E. coli* and purified to homogeneity by affinity chromatography, as judged by SDS-PAGE (Figure 3B).

**FIGURE 4:**
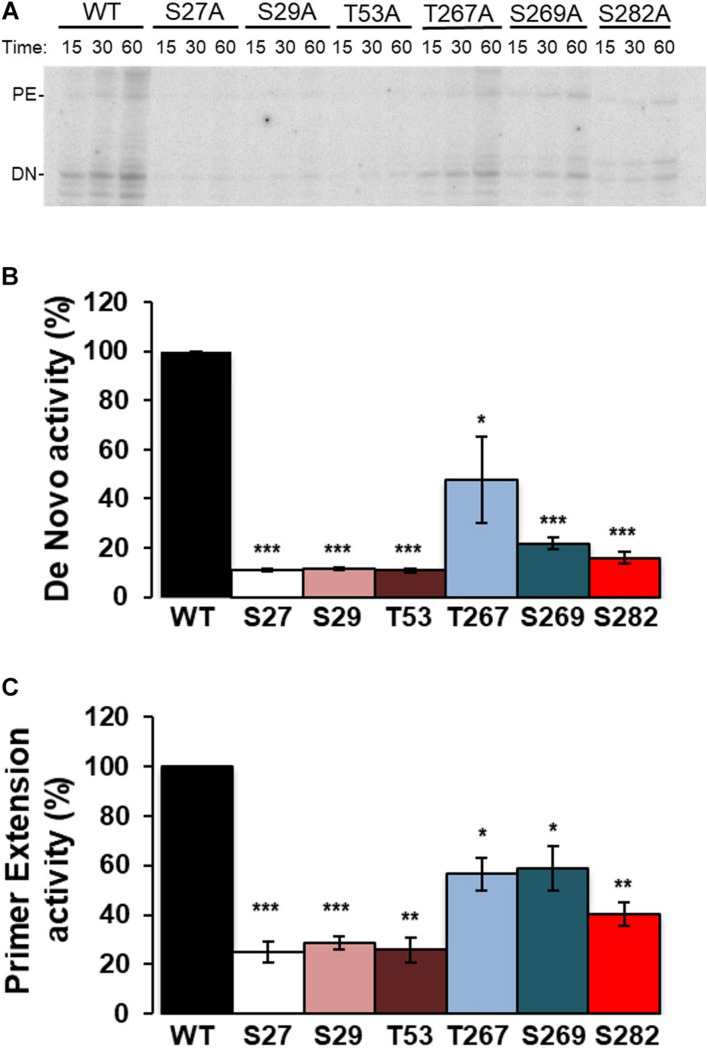
Elongation of LE19 oligonucleotide by *de novo* initiation and primer extension RDRP activity of NS5B Ala mutants. NS5B WT and Ala mutants of residues S27, S29, T53, T267, S269, and S282 were assayed as described in “Materials and Methods” section. Aliquots were stopped at 15, 30, and 60 min, and resolved in denaturing polyacrylamide gels (PAA 23%). **(A)** Representative experiment showing products obtained by primer extension (PE) and *de novo* initiation (DN) using LE19 RNA as a template. Recombinant proteins used are indicated at the top. **(B)** RNA-dependent RNA polymerase *de novo* activity products at 60 min were quantified and represented in arbitrary units. **(C)** RNA-dependent RNA polymerase primer extension activity products at 60 min were quantified and represented in arbitrary units. The mutated residue (Ala mutant) corresponding to each bar is indicated at the bottom. Values in **(B,C)** are the averages (normalized to WT) and corresponding Standard Errors of the Mean (SEM) from at least four independent experiments. Statistically significant differences (Student’s *t*-test) are represented as follows: *p < 0.05; **p < 0.005; ***p < 0.0005. Asterisks over the bar indicate the *p*-value comparing to WT.

**FIGURE 5:**
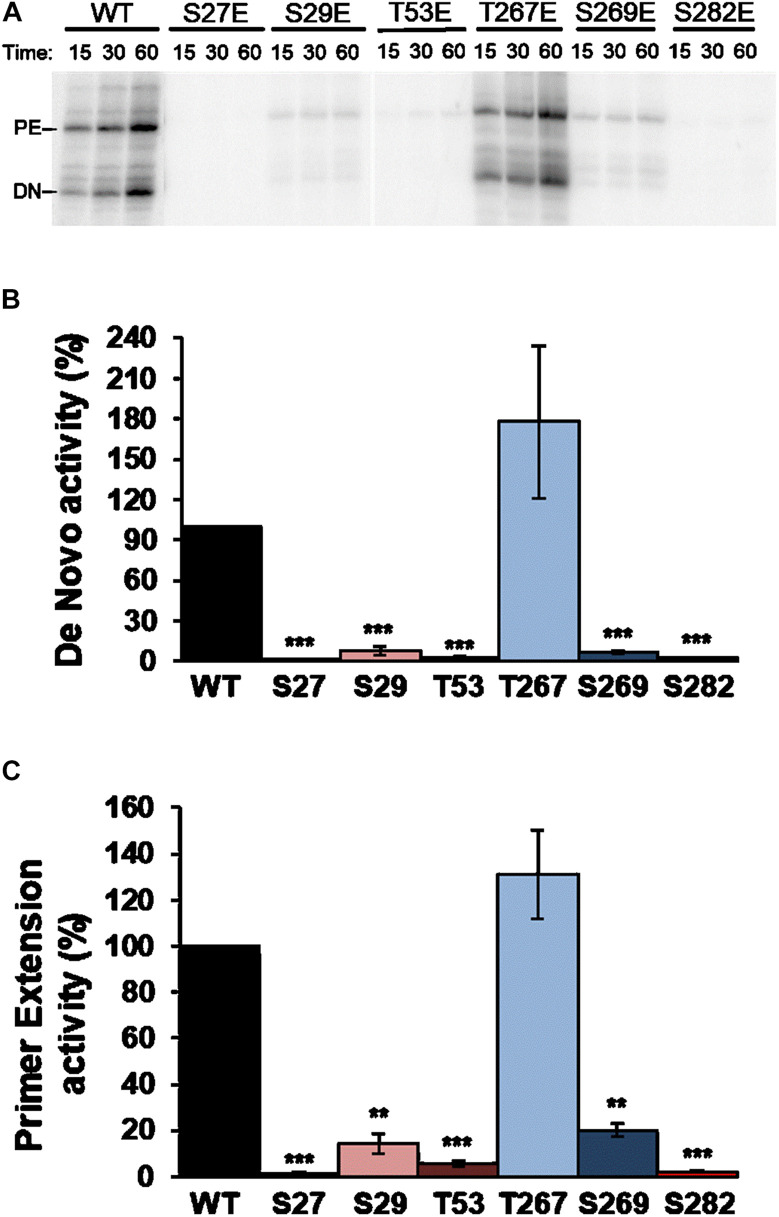
Elongation of LE19 oligonucleotide by *de novo* initiation and primer extension of NS5B Glu mutants. **(A)** Representative experiment showing products obtained by primer extension (PE) and *de novo* initiation (DN) by NS5B WT and mutants imitating phosphorylation (Glu) of residues S27, S29, T53, T267, S269, and S282, using LE19 RNA as a template. Recombinant proteins used are indicated at the top. Quantitative analysis of RNA-dependent RNA polymerase *de novo* **(B)** and primer extension **(C)** activity products at 60 min quantified as described in Figure 4. The mutated residue (Glu mutant) corresponding to each bar is shown at the bottom. Values in **(B,C)** are the averages (normalized to WT) and corresponding Standard Errors of the Mean (SEM) from at least four independent experiments. Statistically significant differences (Student’s *t*-test) are represented as follows: **p < 0.005; ***p < 0.0005. Asterisks over the bar indicate the *p*-value comparing to WT.

RDRP activity assays were carried out using oligonucleotide LE19, which allows *de novo* initiation and primer extension to be analyzed at the same time. Results with Ala mutants showed activity levels below 50% compared to WT in all cases and for both types of activity. Of all the Ala mutants tested, T267A showed the highest activity levels (Figure 4). Assays with the Glu mutants showed imperceptible activity levels for all mutants except T267E, which showed RNA polymerase activity similar to or even higher than the WT protein (Figure 5). Therefore, changes that mimic or abrogate phosphorylation at the NS5B protein positions identified to be phosphorylated by Akt yield proteins with much lower levels of RDRP activity than the WT protein for both *de novo* and primer-dependent synthesis, with the sole exception of the T267E mutant.

**FIGURE 6:**
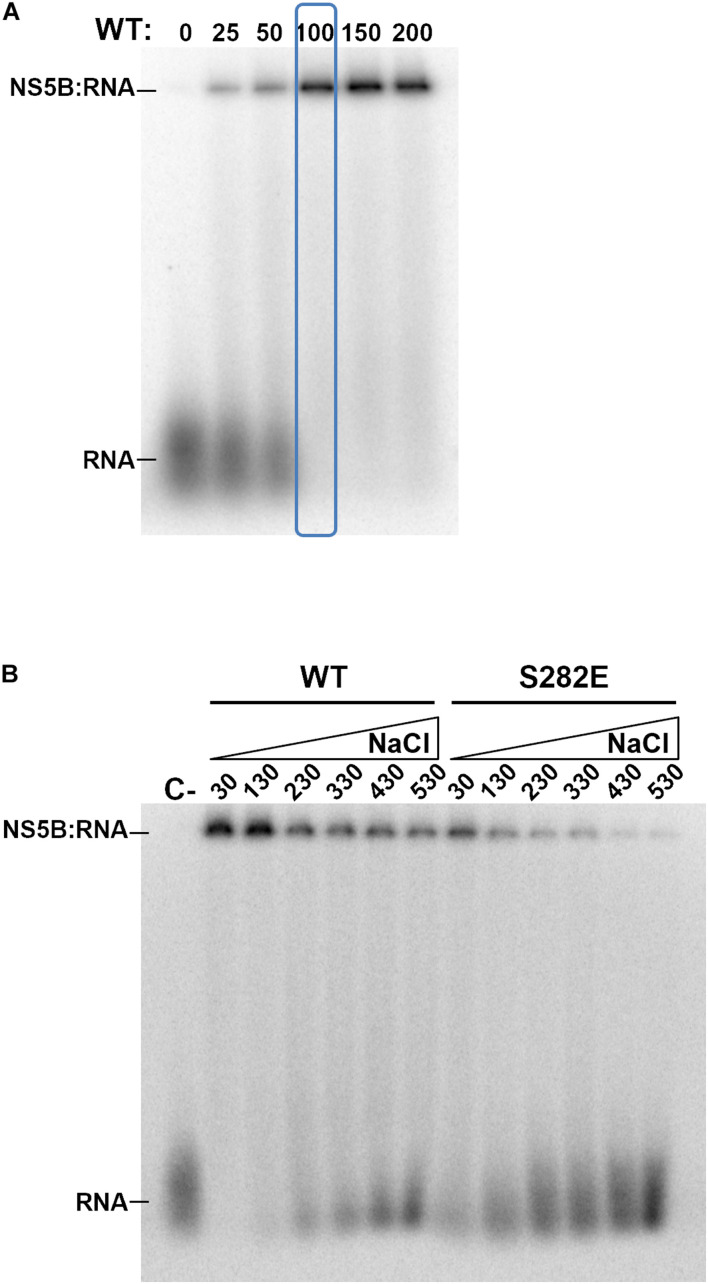
Electromobility shift assays. **(A)** Gel showing retardation of a labeled RNA (10,000 cpm each lane) by increasing concentrations of WT NS5B. Concentration of WT NS5B is indicated at the top (nM). The protein concentration chosen for the following experiments is indicated by a blue box. **(B)** Gel showing retardation of a labeled RNA by WT and S282E NS5B proteins at increasing concentrations (30, 130, 230, 330, 430, and 530 mM) of NaCl. Free labeled probe (RNA) and retarded products (NS5B:RNA) are indicated.

Phosphorylation of Ser and Thr residues leads to the introduction of negative net charges in these positions. Ser282, one of the NS5B residues that becomes phosphorylated by Akt, is part of the active center of the polymerase. We therefore wanted to analyze whether the mutant that mimics phosphorylation in this position maintains the ability to bind RNA. For this purpose, we carried out electromobility shift assay (EMSA) experiments comparing WT and S282E proteins. First, we determined the concentration of WT protein that was able to bind and delay a fixed RNA concentration (corresponding to 10,000 cpm) and at a 100 mM NaCl concentration. The results show that at 100 nM protein all RNA is forming RNA:protein complexes (Figure 6A). Then, with those conditions (10,000 cpm RNA and 100 nM protein) as starting point, we performed electromobility shift assays varying the NaCl concentration to compare WT and S282E proteins. Since RNA:protein interactions are predominantly electrostatic, an increase in ionic strength will result in the loss of interaction. When the experiment was carried out at the lowest NaCl concentration (30 mM), all of the RNA with WT protein is forming RNA:protein complexes (Figure 6B). However, with the protein carrying the S282E mutation under these conditions, a free-form RNA band is observed, indicating less interaction between RNA and mutant protein compared to the WT protein (Figure 6B). In addition, whereas the WT protein even shows an intense band corresponding to RNA: protein complexes at the highest NaCl concentration tested (530 mM), the mutant protein shows very faint complex bands at concentrations higher than 330 mM NaCl (Figure 6B). These results indicate that, under the same conditions, the interaction of RNA with the S282E mutant protein is weaker than with the WT protein.

### Effect of Mutations in T53 and S282 Residues on Virus Replication in Cell Culture

**FIGURE 7:**
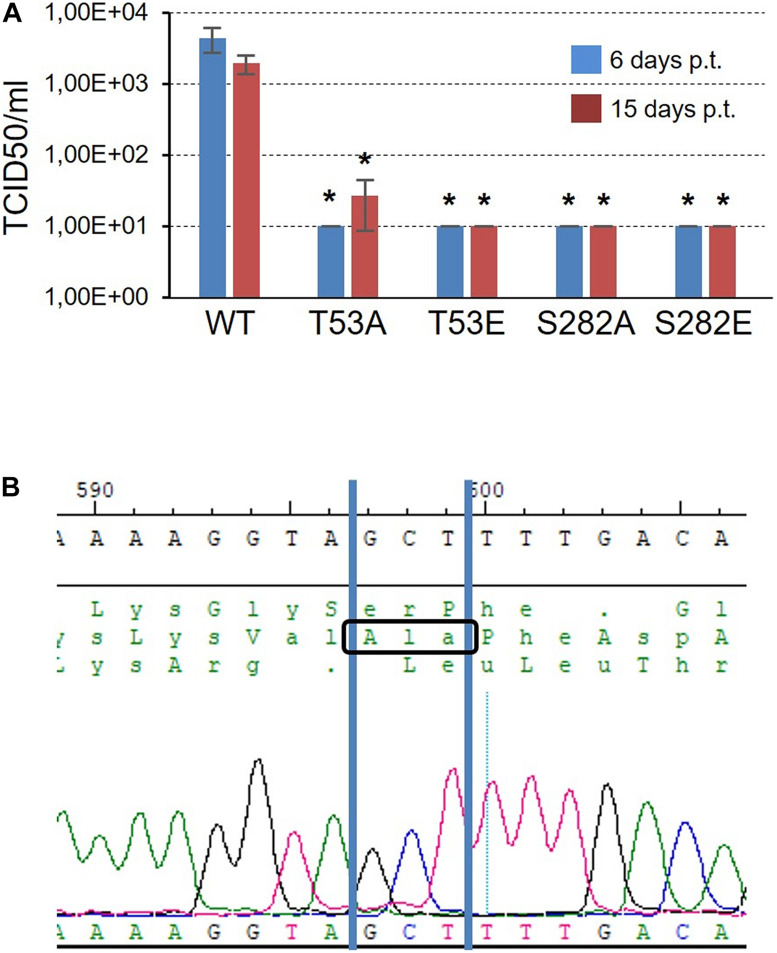
Infectivity of T53 and S282 mutants. **(A)** TCID50/ml values obtained 6 and 15 days post-transfection (blue and red bars, respectively) after transfecting Huh7.5 cells with RNA corresponding to WT virus and the T53A, T53E, S282A, and S282E mutants. The threshold value was established at 10 TCID50/ml. Statistically significant differences (Student’s *t*-test) are represented as follows: *p < 0.05. Asterisk over the bar indicate the *p*-value comparing to WT. **(B)** Chromatogram showing the sequence obtained from the RNA of the T53A mutant rescued 15 days post-transfection. The GCT codon corresponding to the amino acid Ala is identified.

Next, the effect of NS5B phosphorylation by Akt or, alternatively, its inability to completely phosphorylate the polymerase, on HCV’s replicative capacity was analyzed. To do this, we constructed T53A, T53E, S282A, and S282E mutants in the Jc1FLAG2(p7-nsGluc2A) plasmid, the resulting plasmids were transcribed *in vitro*, and the RNA product of the transcription was used to transfect Huh-7.5 cells. The supernatant from these transfected cells was used to titrate for the presence of HCV following a described procedure (Perales et al., 2013; Sheldon et al., 2014). WT virus could be recovered from the supernatant of all three replicates of transfected cells with the corresponding RNA (Figure 7A). The average viral titer was 4.40 × 10^3^ TCID50/ml at 6 days post-transfection, and 1.96 × 10^3^ TCID50/ml at 15 days post-transfection. On the contrary, only two of the mutant virus replicates, both corresponding to the T53A mutant 15 days post-transfection, gave a viral titer value above the cut-off value (Figure 7A). T53A TCID50/ml values were 4.64 × 10^1^ and 2.43 × 10^1^.

To rule out that the virus collected from the supernatant of the cells transfected with the mutant T53A was a product of mutation reversal, we purified the viral RNA, subjected it to an RT-PCR reaction and sequenced the DNA product. The result allowed us to confirm that the T53A change was present in the rescued virus (Figure 7B).

## Discussion

**FIGURE 8:**
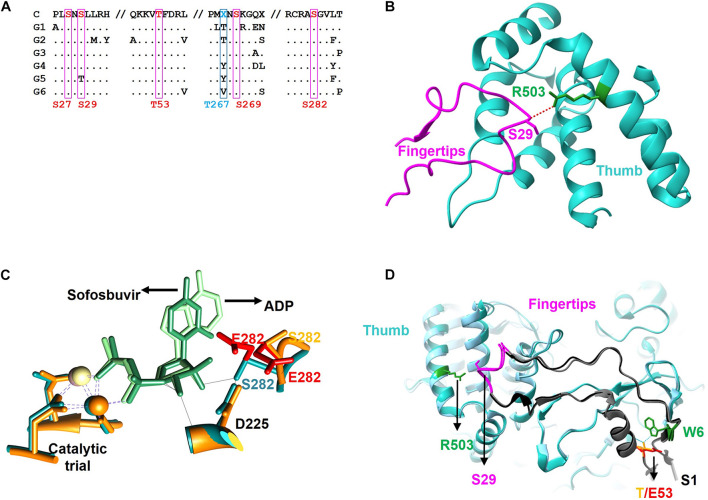
Sequence and structure of the NS5B protein. **(A)** Partial amino acid sequence alignment of the consensus sequences of the major HCV genotypes (G1, G2, G3, G4, G5, and G6) showing the positions described in this work (S27, S29, T53, S269, and S282 in red and T267 in blue) as well as the surrounding residues. The consensus sequence is shown at the top **(C)**. Conserved residues are represented as a dot. **(B)** Zoom of the fingertip region of the NS5B protein showing residues S29 (magenta, fingertip domain) and R503 (blue, thumb domain) and the hydrogen bond connecting them (red dashed line). **(C)** Zoom of the catalytic center of NS5B obtained by aligning the protein structures in the presence of ADP (blue backbone) and sofosbuvir (orange backbone). The catalytic triad coordinated with beta and gamma phosphates of the incoming nucleotide (ADP in light green and sofosbuvir in dark green), and positions D225 and S282 are shown. The most likely rotamers for the S282E mutant are shown in red. **(D)** Zoom showing the Δ1 loop in dark gray. Fingertips with the amino acid S29 and its partner R503 are shown on the left side of the image. On the right are residues S1 (gray), W6 (green) and T53 (orange), as well as the most likely rotamer for the T53E mutant (in red). Hydrogen-bridging bonds between T53 and W6 are shown with dotted blue lines.

Previous work in our laboratory has shown that the cellular kinase Akt interacts with and phosphorylates the HCV polymerase NS5B (Valero et al., 2016). Now, we have identified phosphorylated positions, and analyzed the effect these modifications have on RDRP activity and virus replication. We identified positions S29 (or much less likely S27), T53, S269 (or much less probable T267) and S282 as substrates of the cellular Akt kinase (Figure 1). Coverage of the NS5B proteomic analysis was 70% of the sequence, leaving the possibility that some other phosphorylable positions have not been identified. Previous work has shown that Akt is important for virus replication in HCV-infected cells in culture (13 and references therein), suggesting that the HCV-Akt relationship is important *in vivo* and supporting the *in vitro* results described in the present work. Residue T267 is not conserved among genotypes (Figure 8A), and mutations at this position rendered the highest RDRP activity values among all mutants analyzed in this study, and even better than WT in the case of the T267E mutant. Residue T53 has not been described so far in relation to NS5B activity nor HCV replication. S29 and S282 residues have been previously described as important for both RDRP activity and viral replication. S29 has already been described previously as substrate of other important kinases in the HCV replicative cycle (Han et al., 2014; Hernandez et al., 2015). Furthermore, S29 is the homologous position of T33 of norovirus, which is also phosphorylated by Akt (Eden et al., 2011). These antecedents indicate us that phosphorylation of S29 seems to be very important for the replicative cycle of HCV (Han et al., 2014). Finally, S282 is a residue involved in substrate recognition by interacting with other amino acids (D225) and ribonucleotide substrates (Figure 8B; Appleby et al., 2015). Residue S282 has also been related to sofosbuvir and ribavirin resistance (Aloia et al., 2012; Ji et al., 2015; Kulkarni et al., 2016; Fourati et al., 2019). Therefore, with the exception of position T267, the positions identified in this work that are modified by Akt are critical for the RDRP activity of the NS5B protein as any change greatly affects RDRP activity.

S29 is located in the fingertips subdomain and is part of the residues involved in fingers-thumb interaction (Cai et al., 2005; Figures 3, 8B). Previous *in vitro* work focused on this NS5B region showed that fingers:thumb interactions were key for RDRP activity, binding of RNA and for the transition from *de novo* initiation to primer extension (Chinnaswamy et al., 2008, 2010). In addition, viruses carrying the S29A mutation have serious replication defects in cell culture (Han et al., 2014), which is consistent with the low levels of RDRP activity we have found (Figures 4, 5). The S29E change decreases the distance with R503, a residue that is at binding distance and is also part of the fingers:thumb interaction region (Figure 8B). The distance between S29 and R503 is between 3.3 and 3.8 Å, while the distance between the most stable rotamer of mutant S29E and R503 is between 3.4 and 2.6 Å. In addition, the phosphorylation of S29 introduces a negative charge in that location, and, considering the proximity of the R503 residue (≈3 Å), the interactions of this area could be distorted. This NS5B region must undergo large conformational changes for allowing the transition from *de novo* synthesis to primer extension (Chinnaswamy et al., 2008, 2010; Appleby et al., 2015). Mutations in these positions (e.g., phosphorylation of S29) could prevent these conformational changes or even impair a proper folding of the NS5B protein to carry out the replication of the genome.

Position S282 is part of the NS5B catalytic pocket (Figure 8C). This residue is part of the motif B next to D225 and is essential for the accurate positioning of NTP and the subsequent formation of the phosphodiester bond. The crystalline structure of NS5B in the presence of ADP shows that S282 has a hydrogen bond with the ribose of the nucleotide (Figure 8C; Appleby et al., 2015). By introducing the mutation S282E to mimic the phosphorylation in that position, all the rotamers including the more stable ones lost that hydrogen bond (Figure 8A). Previously, it has been described that changes S282T/G/C/R are associated with resistance to sofosbuvir (Lam et al., 2012; Perales et al., 2015; Chen et al., 2016; Gane et al., 2017). Changes at this position, even the most subtle (S282T) that could be also phosphorylated by Akt, give rise to viruses with very low replication efficiency (Suda et al., 2019; Figure 7). The crystalline structure of the NS5B protein with sofosbuvir located in its active center has been determined (Appleby et al., 2015). This structure showed that S282 is displaced to avoid steric hindrance (compare the protein structures with ADP in cyan and with sofosbuvir in orange in Figure 8C). In addition, the S282E mutation does not allow the hydrogen bond with the ribose ring of the incoming nucleotide in any of the structures, neither in the presence of ADP nor sofosbuvir, as the WT protein does. The lack of an interaction network together with the displacement of protein motifs could be related to the results shown in Figures 4–7.

Position T53 is the least studied of the above-mentioned phosphorylable residues. It is fully conserved in all HCV genotypes. The amino acid T53 is probably involved in maintaining the structure of the Δ1 loop and fingertips through interactions with other amino acids such as W6, with which it establishes hydrogen bonds (Figure 8D). Other hydrogen bonds involved would be those formed by residues S1:R56, M2:R56, S3:V52, S3:F54, and E17:S42. Notably, the Δ1 loop S42 residue is a substrate of the serine kinase PRK2, and mutations at this position also affect RDRP activity and HCV replication (Han et al., 2014). Therefore, phosphorylation of S42 and T53, together with S29, could be destabilizing the interactions that allow the maintenance of an active NS5B structure. The large number of interactions between positions 1–6 and 52–56 could be responsible for maintaining the structure of this region of the NS5B protein and thus for the replication of the T53A mutant (Figure 7), albeit at much lower levels than the parental virus.

In summary, the positions described in this work are phosphorylated *in vitro* by Akt, a Ser/Thr kinase involved in many cellular processes. The positions identified are critical for NS5B RDRP activity, and Ser/Thr to Glu changes at these positions (imitating phosphorylation) as well as changes to Ala result in loss of HCV polymerase activity. Furthermore, mutants that mimic phosphorylation are unable to replicate in cell culture. The reason why HCV polymerase is a substrate for cellular kinases is currently unclear. The inactive NS5B protein product of phosphorylation might be able to interact with other viral or cellular proteins, thus modulating the viral and cellular replicative cycle. These lines of research are currently being explored in our laboratory.

## Data Availability Statement

The raw data supporting the conclusions of this article will be made available by the authors, without undue reservation.

## Author Contributions

RS and AM: conceptualization, formal analysis, and writing—original draft preparation. RS, LA-G, BP, PC-C, ER, CG-C, IG, AÁ, and AM: investigation. RS, CP, ED, and AM: methodology. RS, LA-G, PC-C, and AM: writing—review and editing. CP, ED, and AM: funding acquisition. All authors contributed to the article and approved the submitted version.

## Conflict of Interest

The authors declare that the research was conducted in the absence of any commercial or financial relationships that could be construed as a potential conflict of interest.

## Publisher’s Note

All claims expressed in this article are solely those of the authors and do not necessarily represent those of their affiliated organizations, or those of the publisher, the editors and the reviewers. Any product that may be evaluated in this article, or claim that may be made by its manufacturer, is not guaranteed or endorsed by the publisher.

## References

[B1] Albentosa-Gonzalez L. Sabariegos R. Arias A. Clemente-Casares P. Mas A. (2021). Akt interacts with usutu virus polymerase, and its activity modulates viral replication. *Pathogens* 10:244. 10.3390/pathogens10020244 33672588 PMC7924047

[B2] Aloia A. L. Locarnini S. Beard M. R. (2012). Antiviral resistance and direct-acting antiviral agents for HCV. *Antivir Ther.* 17(6 Pt B), 1147–1162. 10.3851/imp2426 23188771

[B3] Appleby T. C. Perry J. K. Murakami E. Barauskas O. Feng J. Cho A. (2015). Viral replication. Structural basis for RNA replication by the hepatitis C virus polymerase. *Science* 347 771–775. 25678663 10.1126/science.1259210

[B4] Barik S. McLean T. Dupuy L. C. (1995). Phosphorylation of Ser232 directly regulates the transcriptional activity of the P protein of human respiratory syncytial virus: phosphorylation of Ser237 may play an accessory role. *Virology* 213 405–412. 10.1006/viro.1995.0013 7491765

[B5] Cai Z. Yi M. Zhang C. Luo G. (2005). Mutagenesis analysis of the rGTP-specific binding site of hepatitis C virus RNA-dependent RNA polymerase. *J. Virol.* 79 11607–11617. 10.1128/jvi.79.18.11607-11617.2005 16140738 PMC1212605

[B6] Chen Z. W. Li H. Ren H. Hu P. (2016). Global prevalence of pre-existing HCV variants resistant to direct-acting antiviral agents (DAAs): mining the GenBank HCV genome data. *Sci. Rep.* 6:20310. 10.1038/srep20310 PMC4740856 26842909

[B7] Chinnaswamy S. Murali A. Li P. Fujisaki K. Kao C. C. (2010). Regulation of de novo-initiated RNA synthesis in hepatitis C virus RNA-dependent RNA polymerase by intermolecular interactions. *J. Virol.* 84 5923–5935. 10.1128/jvi.02446-09 20375156 PMC2876623

[B8] Chinnaswamy S. Yarbrough I. Palaninathan S. Kumar C. T. Vijayaraghavan V. Demeler B. (2008). A locking mechanism regulates RNA synthesis and host protein interaction by the hepatitis C virus polymerase. *J. Biol. Chem.* 283 20535–20546. 10.1074/jbc.m801490200 18442978 PMC2459299

[B9] Clemente-Casares P. Lopez-Jimenez A. J. Bellon-Echeverria I. Encinar J. A. Martinez-Alfaro E. Perez-Flores R. (2011). De novo polymerase activity and oligomerization of hepatitis C virus RNA-dependent RNA-polymerases from genotypes 1 to 5. *PLoS One* 6:e18515. 10.1371/journal.pone.0018515 21490973 PMC3072391

[B10] de Chassey B. Navratil V. Tafforeau L. Hiet M. S. Aublin-Gex A. Agaugue S. (2008). Hepatitis C virus infection protein network. *Mol. Syst. Biol.* 4:230. 10.1038/msb.2008.66 PMC2600670 18985028

[B11] Dolan P. T. Zhang C. Khadka S. Arumugaswami V. Vangeloff A. D. Heaton N. S. (2013). Identification and comparative analysis of hepatitis C virus-host cell protein interactions. *Mol. Biosyst.* 9 3199–3209. 10.1039/c3mb70343f 24136289 PMC4171131

[B12] Eden J. S. Sharpe L. J. White P. A. Brown A. J. (2011). Norovirus RNA-dependent RNA polymerase is phosphorylated by an important survival kinase. *Akt. J. Virol.* 85 10894–10898. 10.1128/jvi.05562-11 21849454 PMC3187498

[B13] Fourati S. Rodriguez C. Hezode C. Soulier A. Ruiz I. Poiteau L. (2019). Frequent antiviral treatment failures in patients infected with hepatitis C virus genotype 4. Subtype 4r. *Hepatology* 69 513–523. 10.1002/hep.30225 30125371

[B14] Gane E. J. Metivier S. Nahass R. Ryan M. Stedman C. A. Svarovskaia E. S. (2017). The emergence of NS5B resistance associated substitution S282T after sofosbuvir-based treatment. *Hepatol. Commun.* 1 538–549. 10.1002/hep4.1060 29404477 PMC5678900

[B15] Hagen N. Bayer K. Rosch K. Schindler M. (2014). The intraviral protein interaction network of hepatitis C virus. *Mol. Cell Proteomics* 13 1676–1689. 10.1074/mcp.m113.036301 24797426 PMC4083108

[B16] Hamamoto I. Nishimura Y. Okamoto T. Aizaki H. Liu M. Mori Y. (2005). Human VAP-B is involved in hepatitis C virus replication through interaction with NS5A and NS5B. *J. Virol.* 79 13473–13482. 10.1128/jvi.79.21.13473-13482.2005 16227268 PMC1262604

[B17] Han S. H. Kim S. J. Kim E. J. Kim T. E. Moon J. S. Kim G. W. (2014). Phosphorylation of hepatitis C virus RNA polymerases ser29 and ser42 by protein kinase C-related kinase 2 regulates viral RNA replication. *J. Virol.* 88 11240–11252. 10.1128/jvi.01826-14 25031343 PMC4178806

[B18] Hernandez S. Figueroa D. Correa S. Diaz A. Aguayo D. Villanueva R. A. (2015). Phosphorylation at the N-terminal finger subdomain of a viral RNA-dependent RNA polymerase. *Biochem. Biophys. Res. Commun.* 466 21–27. 10.1016/j.bbrc.2015.08.082 26301630

[B19] Hillung J. Ruiz-Lopez E. Bellon-Echeverria I. Clemente-Casares P. Mas A. (2012). Characterization of the interaction between hepatitis C virus NS5B and the human oestrogen receptor alpha. *J. Gen. Virol.* 93(Pt 4), 780–785. 10.1099/vir.0.039396-0 22170636

[B20] Inoue Y. Aizaki H. Hara H. Matsuda M. Ando T. Shimoji T. (2011). Chaperonin TRiC/CCT participates in replication of hepatitis C virus genome via interaction with the viral NS5B protein. *Virology* 410 38–47. 10.1016/j.virol.2010.10.026 21093005

[B21] Jakubiec A. Tournier V. Drugeon G. Pflieger S. Camborde L. Vinh J. (2006). Phosphorylation of viral RNA-dependent RNA polymerase and its role in replication of a plus-strand RNA virus. *J. Biol. Chem.* 281 21236–21249. 10.1074/jbc.m600052200 16717096

[B22] Ji H. Kozak R. A. Biondi M. J. Pilon R. Vallee D. Liang B. B. (2015). Next generation sequencing of the hepatitis C virus NS5B gene reveals potential novel S282 drug resistance mutations. *Virology* 477 1–9. 10.1016/j.virol.2014.12.037 25600207

[B23] Kulkarni A. S. Damha M. J. Schinazi R. F. Mo H. Doehle B. Sagan S. M. (2016). A complex network of interactions between S282 and G283 of hepatitis C virus nonstructural protein 5b and the template strand affects susceptibility to sofosbuvir and ribavirin. *Antimicrob Agents Chemother.* 60 2018–2027. 10.1128/aac.02436-15 26824949 PMC4808174

[B24] Kusakawa T. Shimakami T. Kaneko S. Yoshioka K. Murakami S. (2007). Functional interaction of hepatitis C Virus NS5B with Nucleolin GAR domain. *J. Biochem.* 141 917–927. 10.1093/jb/mvm102 17569707

[B25] Lam A. M. Espiritu C. Bansal S. Micolochick Steuer H. M. Niu C. Zennou V. (2012). Genotype and subtype profiling of PSI-7977 as a nucleotide inhibitor of hepatitis C virus. *Antimicrob Agents Chemother.* 56 3359–3368. 10.1128/aac.00054-12 22430955 PMC3370800

[B26] Liu Z. Tian Y. Machida K. Lai M. M. Luo G. Foung S. K. (2012). Transient activation of the PI3K-AKT pathway by hepatitis C virus to enhance viral entry. *J. Biol. Chem.* 287 41922–41930. 10.1074/jbc.m112.414789 23095753 PMC3516739

[B27] Lopez-Jimenez A. J. Clemente-Casares P. Sabariegos R. Llanos-Valero M. Bellon-Echeverria I. Encinar J. A. (2014). Hepatitis C virus polymerase-polymerase contact interface: significance for virus replication and antiviral design. *Antiviral Res.* 108 14–24. 10.1016/j.antiviral.2014.04.009 24815023

[B28] Munakata T. Nakamura M. Liang Y. Li K. Lemon S. M. (2005). Down-regulation of the retinoblastoma tumor suppressor by the hepatitis C virus NS5B RNA-dependent RNA polymerase. *Proc. Natl. Acad. Sci. U S A.* 102 18159–18164. 10.1073/pnas.0505605102 16332962 PMC1307512

[B29] Neufeldt C. J. Cortese M. Acosta E. G. Bartenschlager R. (2018). Rewiring cellular networks by members of the flaviviridae family. *Nat. Rev. Microbiol.* 16 125–142. 10.1038/nrmicro.2017.170 29430005 PMC7097628

[B30] Perales C. Beach N. M. Gallego I. Soria M. E. Quer J. Esteban J. I. (2013). Response of hepatitis C virus to long-term passage in the presence of alpha interferon: multiple mutations and a common phenotype. *J. Virol.* 87 7593–7607. 10.1128/jvi.02824-12 23637397 PMC3700284

[B31] Perales C. Quer J. Gregori J. Esteban J. I. Domingo E. (2015). Resistance of hepatitis c virus to inhibitors: complexity and clinical implications. *Viruses* 7 5746–5766. 10.3390/v7112902 26561827 PMC4664975

[B32] Qadri I. Choudhury M. Rahman S. M. Knotts T. A. Janssen R. C. Schaack J. (2012). Increased phosphoenolpyruvate carboxykinase gene expression and steatosis during hepatitis C virus subgenome replication: role of nonstructural component 5A and CCAAT/enhancer-binding protein beta. *J. Biol. Chem.* 287 37340–37351. 10.1074/jbc.m112.384743 22955269 PMC3481331

[B33] Qian X. Xu C. Wu B. Tang H. Zhao P. Qi Z. (2020). SNORD126 promotes hepatitis C virus infection by upregulating Claudin-1 via activation of PI3K-AKT signaling pathway. *Front. Microbiol.* 11:565590. 10.3389/fmicb.2020.565590 33042070 PMC7522514

[B34] Schmid S. Mayer D. Schneider U. Schwemmle M. (2007). Functional characterization of the major and minor phosphorylation sites of the P protein of Borna disease virus. *J. Virol.* 81 5497–5507. 10.1128/jvi.02233-06 17376920 PMC1900310

[B35] Sesmero E. Thorpe I. F. (2015). Using the hepatitis C virus RNA-Dependent RNA polymerase as a model to understand viral polymerase structure. *Funct. Dynam. Viruses* 7 3974–3994. 10.3390/v7072808 26193306 PMC4517137

[B36] Sheldon J. Beach N. M. Moreno E. Gallego I. Pineiro D. Martinez-Salas E. (2014). Increased replicative fitness can lead to decreased drug sensitivity of hepatitis C virus. *J. Virol.* 88 12098–12111. 10.1128/jvi.01860-14 25122776 PMC4178724

[B37] Suda G. Kimura M. Shigesawa T. Suzuki K. Nakamura A. Ohara M. (2019). Effects of resistance-associated variants in genotype 2 hepatitis C virus on viral replication and susceptibility to antihepatitis C virus drugs. *Hepatol. Res.* 49 1275–1285. 10.1111/hepr.13401 31261439

[B38] Tabata K. Neufeldt C. J. Bartenschlager R. (2020). Hepatitis C virus replication. *Cold Spring Harb. Perspect. Med.* 10:a037093. 10.1101/cshperspect.a037093 PMC7050578 31570388

[B39] Valero M. L. Sabariegos R. Cimas F. J. Perales C. Domingo E. Sanchez-Prieto R. (2016). Hepatitis C virus RNA-Dependent RNA polymerase interacts with the Akt/PKB kinase and induces its subcellular relocalization. *Antimicrob Agents Chemother* 60 3540–3550. 10.1128/aac.03019-15 27021315 PMC4879427

[B40] Watashi K. Ishii N. Hijikata M. Inoue D. Murata T. Miyanari Y. (2005). Cyclophilin B is a functional regulator of hepatitis C virus RNA polymerase. *Mol. Cell* 19 111–122. 10.1016/j.molcel.2005.05.014 15989969

[B41] Zhang H. Zhang C. Tang H. Gao S. Sun F. Yang Y. (2018). CD2-Associated protein contributes to hepatitis C, virus propagation and steatosis by disrupting insulin signaling. *Hepatology* 68 1710–1725. 10.1002/hep.30073 29729186 PMC6220802

